# Metropolitan Hospital Sunday Fund

**Published:** 1898-08-27

**Authors:** 


### The Institutional Workshop.

### METROPOLITAN HOSPITAL SUNDAY FUND.

At the annual meeting of the Metropolitan Hospital Sunday Fund 1898, it was reported that the total amount collected to the date of the meeting was £38,741. It has always been the wish of the Council to raise at least £50,000 per annum, although, so far, the average yield has remained about £40,000 a year. Sir Sydney Waterlow gave it as his opinion that the sum raised must be considered satisfactory, taking into account the various difficulties against which the Fund had to contend. It was further pointed out that, owing, no doubt, in a measure to a desire not to harass the clergy and ministers of religion, no effort of any kind had been put forth by the Council to call special attention to collections on Hospital Sunday during 1898. We are confident that a central fund like the Hospital Sunday Fund, in order to succeed, must be administered with continuous energy or it must necessarily fail to attract attention, and so to win adequate public support. We are strongly of opinion that, at the meeting of the constituents of the Fund at the end of the present year, it would be well that a resolution should be brought forward urging the Council to institute special efforts on behalf of the Hospital Sunday Fund during 1899, and to concert measures whereby more publicity will each year be given to the collections through the Press.

The history of the Hospital Sunday Fund shows that whenever the Council has put forward an effort of this kind it has resulted in a large increase in the amount of the collections, and that such increase has been maintained in the years succeeding that in which they were put forth. Thus, in 1883 the Fund fell to less than £34,000, a circumstance which so moved the late Dr. James Wakley that he gave a special donation of £1,000, and was the means of inducing a special effort to be made which brought the collections up to nearly £40,000 in 1886. From that year to 1893 the average of the Fund was about £42,000 per annum. In 1893 and 1894 the Fund, inclusive of the extraordinary contributions, fell below £40,000, due to a diminution in the amounts received from the congregations. In 1895, owing to special efforts, the Fund was increased to the record sum of upwards of £60,000. The congregational collections in that year also amounted to the largest sum received up to this date from this source. In 1896, owing to a special letter addressed to the clergy and ministers by the Lord Mayor, the amount received from the congregations increased to £40,500, the largest sum ever collected in the churches of the metropolis. In 1897 the congregational collections fell away to a little over £37,000, and the present year shows a further reduction.

It thus appears that the present financial position of the Fund is similar to what it was in 1885, in 1893, and 1894, and we have no doubt that a little more energy and effort would be well expended in 1898-9, with the result that it will then be shown that the giving power of the congregations has not only not diminished, but that it is capable of being considerably increased. The knowledge of these facts induces us to urge Sir Sydney Waterlow to bring forward a resolution at the meeting of the constituents in December urging the Council to renewed efforts on behalf of the hospitals. Heretofore very little money has been spent upon advertisements, and the time has arrived when a new policy should be instituted and continued with a view to maintaining the Fund at a sum worthy of the metropolis and equal to the needs of the hospitals.

Incidentally it was pointed out that the substitution of limited liability companies for private firms has had a material effect upon the amount of donations which are given to charity in London in the present day. Twenty years ago the number of private bankers and firms transacting enormous business in the City of London was considerable, but every year they have been disappearing, owing to the growth of the limited liability system. It cannot be too widely recognised that an incorporated company has responsibilites in regard to the public institutions and the charities in the particular locality in which its central office is situated. This obligation is generally recognised in the articles of association, and we are inclined to think that if the matter is kept before the shareholders and the directors they will be disposed in increasing numbers to avail themselves of the powers which they possess to give a moderate subscription to the hospitals and to a few other institutions which minister to the requirements of the whole of the population. We do not follow Sir Sydney Waterlow in his contention that the shareholders who uphold directors in making these grants are voting away other people's money, seeing that all profits are the property of the shareholders, and that they can distribute them as they think best. The idea that a grant of £100 by a limited liability company to the Hospital Sunday Fund or the Prince of Wales's Fund, or to one of the great hospitals, will give the individual shareholders the comfortable idea that they have done their duty, and so disposed of any personal obligation to give as such, is in our view unreal and inaccurate. We have had an opportunity of looking into the limited liability companies which have availed themselves of their powers to make contributions of the kind we are discussing, and we find that the shareholders and directors of these companies belong to the class of most liberal givers as individuals. Further, it is the fact that these individual directors and shareholders have in late years, while sanctioning contributions of a reasonable amount for charity from the limited liability undertakings with which they are connected, also increased considerably their individual gifts. For these reasons we are strongly of opinion that concerted measures should be taken to bring the duty of giving to the notice of those who are responsible for the management of the companies and corporations, and especially those which have taken the place of the large private firms who in former years were the mainstay of metropolitan charity. At any rate, it is not justifiable to sit still with folded hands and to shut one's eyes to the altered circumstances of the time. All who have the welfare of the hospitals at heart will agree that a policy of inaction must be given up in favour of measures which will demonstrate that no effort has been spared to bring the claims of the hospitals to the notice of everybody who is in a position to give.

